# An ambient temperature collection and stabilization strategy for canine microbiota studies

**DOI:** 10.1038/s41598-020-70232-6

**Published:** 2020-08-07

**Authors:** Ching-Yen Lin, Tzu-Wen L. Cross, Evgueni Doukhanine, Kelly S. Swanson

**Affiliations:** 1 Division of Nutritional Sciences, University of Illinois at Urbana-Champaign, Urbana, IL 61801 USA; 2 DNA Genotek, Inc., Ottawa, ON Canada; 3 Department of Animal Sciences, University of Illinois at Urbana-Champaign, Urbana, IL 61801 USA

**Keywords:** Applied microbiology, Metagenomics

## Abstract

Similar to humans, the fecal microbiome of dogs may be useful in diagnosing diseases or assessing dietary interventions. The accuracy and reproducibility of microbiome data depend on sample integrity, which can be affected by storage methods. Here, we evaluated the ability of a stabilization device to preserve canine fecal samples under various storage conditions simulating shipping in hot or cold climates. Microbiota data from unstabilized samples stored at room temperature (RT) and samples placed in PERFORMAbiome·GUT collection devices (PB-200) (DNA Genotek, Inc. Ottawa, Canada) and stored at RT, 37 °C, 50 °C, or undergoing repeated freeze–thaw cycles, were compared with freshly extracted samples. Alpha- and beta diversity indices were not affected in stabilized samples, regardless of storage temperature. Unstabilized samples stored at RT, however, had higher alpha diversity. Moreover, the relative abundance of dominant bacterial phyla (Firmicutes, Fusobacteria, Bacteriodetes, and Actinobacteria) and 24 genera were altered in unstabilized samples stored at RT, while microbiota abundance was not significantly changed in stabilized samples stored at RT. Our results suggest that storage method is important in microbiota studies and that the stabilization device may be useful in maintaining microbial profile integrity, especially for samples collected off-site and/or those undergoing temperature changes during shipment or storage.

## Introduction

The canine gastrointestinal (GI) tract harbors a highly dense and diverse population of microorganisms, including bacteria, fungi, archaea, and viruses, collectively termed microbiota. In recent years, DNA-based, high-throughput technologies have allowed a better characterization of the GI microbiome and its role in host health and disease. A large number of studies have demonstrated that altered gut microbiota composition and functions are associated with several metabolic disorders, such as obesity^1,2^, diabetes^3,4^, inflammatory bowel diseases^5–7^, autoimmune diseases^8,9^ and cancers^10,11^ in humans and/or dogs. Because microbial shifts are often noted with disease, microbiome profiles may be used as biomarkers for diagnosis and/or monitoring in the future^12–14^.

The microbiome field has much to offer, but appropriate sample collection and storage methods are needed for sample integrity and accurate and reproducible data. It has been shown that microbiome profiles of human fecal samples are changed after 48 hours of storage at ambient temperatures^15,16^. Many studies also demonstrated that proper stabilization and storage methods are key to having accurate and reproducible microbiome data^15,17–21^. To date, the most appropriate and widely used collection protocol is to rapidly freeze and store samples at − 80° or − 20 °C^17,21,22^. However, these methods may not be practical in many cases. For example, very low-temperature storage might not always be accessible when collecting fecal samples from study subjects at home or in remote locations. Although studies have reported that storing samples at 4 °C slows bacterial growth and maintains sample integrity, they must be processed or stored at lower temperatures within 24 hours^21,23^. In addition, maintaining samples at low temperatures during shipment may be challenging and costly. Therefore, development of strategies able to stabilize samples at ambient temperature is of great interest.

Previous studies used a commercially available ambient temperature stabilization device OMNIgene•GUT (DNA Genotek, Inc. Ottawa, Canada) to collect and store human fecal samples. Results suggested that this device maintained the integrity of microbiome data and allowed for a higher recovery of nucleic acids at room temperature when compared with freezing at − 20 °C^23–25^. Similar to humans, researchers are interested in identifying the relationship between the GI microbiota, nutritional status, and health of dogs. The number of studies of the dog microbiome has grown in recent years^26–28^. There is, however, limited information about canine fecal sample collection and storage methods.

**Figure 1:**
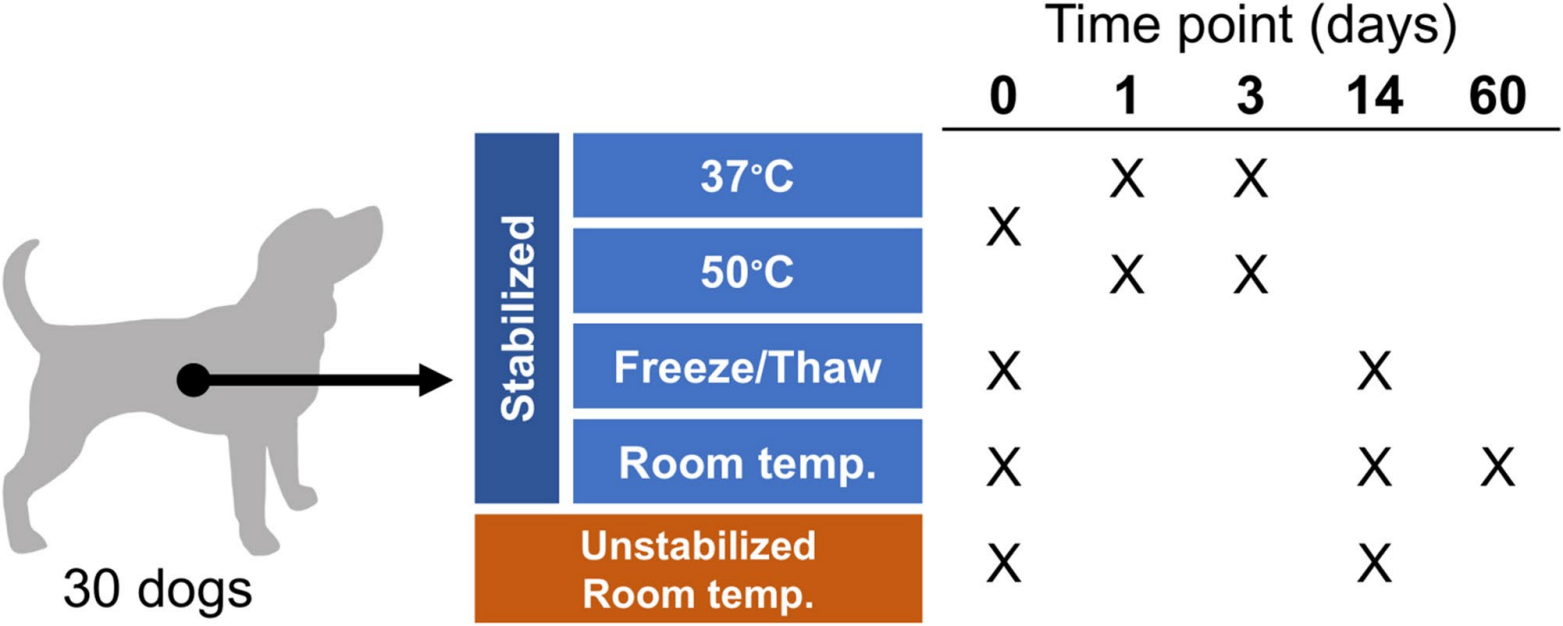
Experimental design. Fresh fecal samples were collected from 30 healthy beagles. Each sample was aliquoted into three stabilization devices and one unstabilized aliquot, and incubated under different conditions. One stabilized sample was aliquoted and incubated at 37 °C and 50 °C after baseline microbiota data were obtained. Microbiota data were obtained at different time points as marked.

The microbiota communities of humans and dogs are distinct. The human gut is dominated by Bacteroidetes and Firmicutes^29,30^ while the canine gut is co-dominated by Bacteroidetes, Firmicutes and Fusobacterium^6,31^. Here, we used a similar commercial stabilization device designed specifically for animals, PERFORMAbiome•GUT (DNA Genotek, Inc. Ottawa, Canada), to study canine fecal sample collection and storage. In addition to what was conducted in previous human studies^23–25^, the current study investigated the effectiveness of this device in preserving samples during different storage temperatures that was meant to mimic shipment conditions where temperatures often change. The effects of a longer storage time (60 days in this study; up to 28 days in human studies) at room temperature  (RT) were also studied. To assess the ability of this device to preserve fecal samples in different conditions, stabilized samples were stored at (1) RT (~ 23 °C), (2) 37 °C, (3) 50 °C, or (4) underwent repeated freeze–thaw (-20 °C/30 °C) cycles. We compared samples extracted on the collection day (baseline) to stabilized samples incubated for 1, 3, 14 or 60 days. Unstabilized samples were also collected and stored at RT to compare with stabilized samples stored at RT (Fig. 1). Finally, in order to determine intra-sampling variation, we compared samples collected from three locations of each fecal sample.

## Results

### Microbiome profiles are largely unaffected by storage method at baseline

**Figure 2:**
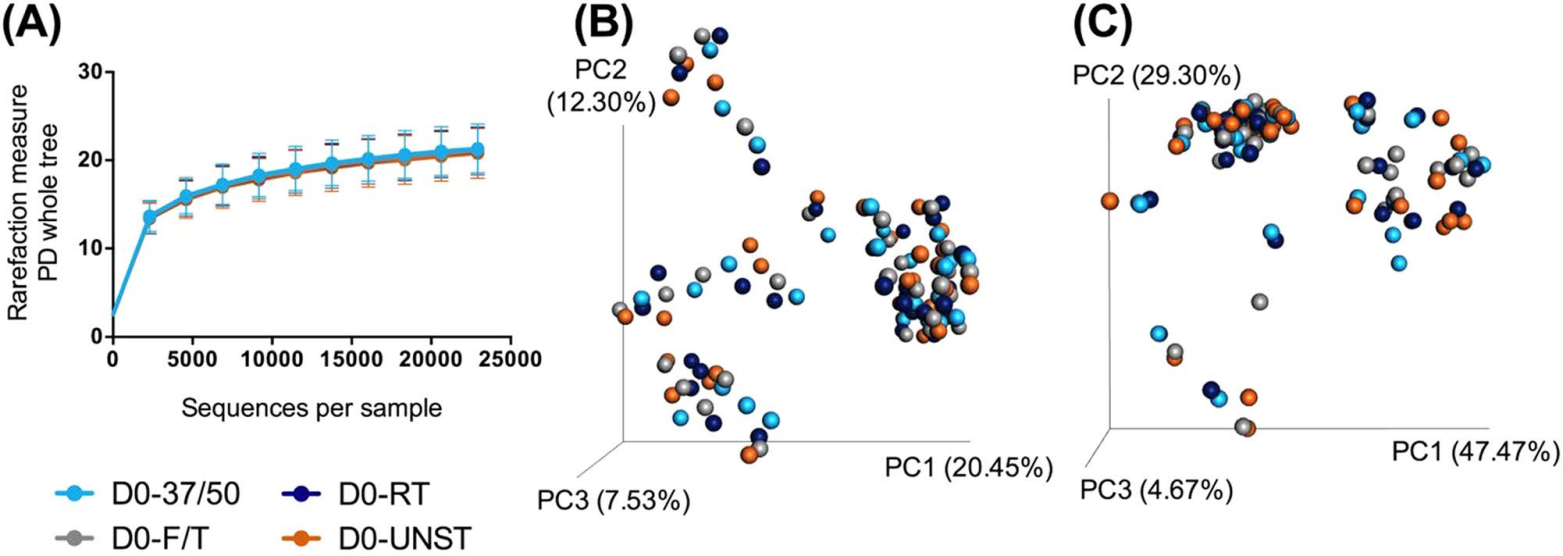
Fecal microbiota communities of baseline stabilized samples (D0-37/50, D0-FT, D0-RT) and unstabilized sample (D0-UNST). (**A**) Alpha diversity measures, including phylogenetic diversity (PD) whole tree (shown), suggested that species richness and diversity were not affected by collection method at baseline (ANOVA, *p* = 0.506). Principal coordinates analysis plots of unweighted (**B**) and weighted (**C**) UniFrac distances of fecal microbial communities revealed that beta diversity was not altered by collection method at baseline.

All baseline samples were extracted on the collection day within 2 hours, including three stabilized samples (D0-37/50, D0-FT, D0-RT) and one unstabilized sample (D0-UNST) were compared. The stabilized samples were from one defecation and were not handled differently. An even sampling depth of 22,931 sequences per sample was used for assessing alpha- and beta diversity measures. Alpha- (*p* = 0.506) and beta diversity measures were not different between baseline samples on the collection day (D0) (Fig. 2). Relative abundances of bacteria at the phylum level were not different (*p* > 0.05) between samples. At the genus level, the relative abundances of *Faecalibacterium* (*p* = 0.001; stabilized: 1.10%; UNST: 0.57%) and *Phascolarctobacterium* (*p* < 0.001; stabilized: 2.74%; UNST: 1.14%) were greater, while relative abundances of *Lactobacillus* (*p* = 0.047; stabilized: 1.70%; UNST: 3.31%) and *Clostridium* (*p* = 0.004; stabilized: 0.77%; UNST: 1.17%) were lower in stabilized samples (D0-37/50, D0-FT, D0-RT) compared with unstabilized samples (D0-UNST). No other differences to taxa were observed.

### Stabilized samples stored at high temperatures yield consistent microbiota profiles that are similar to baseline samples

**Figure 3:**
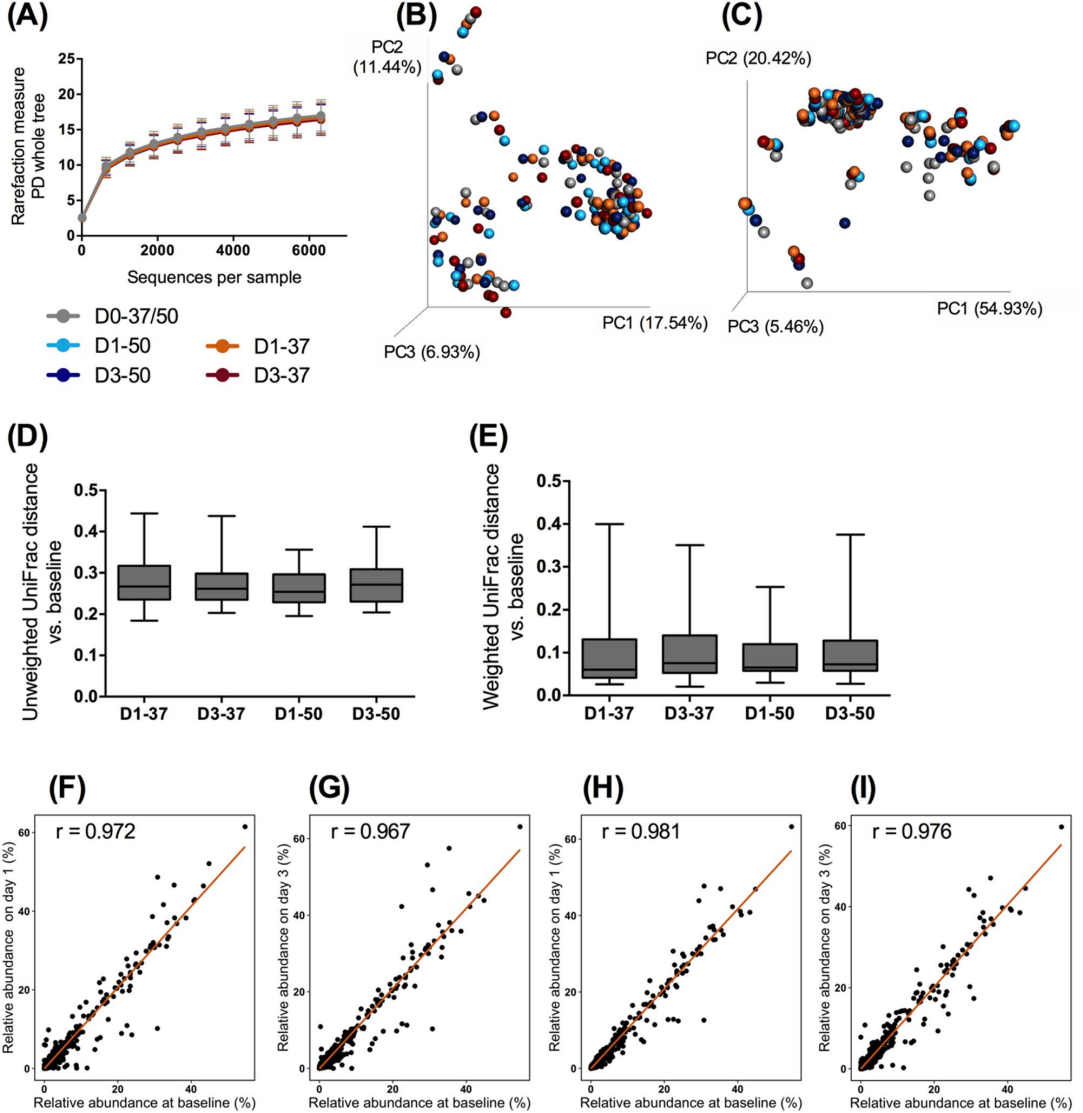
Fecal microbiota communities of stabilized samples at baseline (D0-37/50) and stored at 37 °C and 57 °C for 1 (D1-37, D1-50) and 3 days (D3-37, D3-50). (**A**) Alpha diversity measures, including phylogenetic diversity (PD) whole tree (shown), suggested that species richness and diversity were not affected by storage condition (ANOVA, *p* = 0.944). Principal coordinates analysis plots of unweighted (**B**) and weighted (**C**) UniFrac distances of fecal microbial communities as well as unweighted (**D**) and weighted (**E**) UniFrac distances from baseline revealed that beta diversity was not altered by storage condition. Scatterplots show the relative abundance of each genus in baseline samples against samples stored at 37 °C for 1 (**F**) and 3 days (**G**), and samples stored at 50 °C for 1 (**H**) and 3 days (**I**).

To validate the ability of the stabilization device to preserve samples at high temperatures, which could occur during shipment, stabilized samples were stored at 37 °C or 50 °C for 1 day or 3 days (D1-37, D3-37, D1-50, D3-50, respectively) and compared to baseline samples (D0-37/50). An even sampling depth of 6,310 sequences per sample was used for evaluating alpha- and beta diversity measures. Alpha- (*p* = 0.944) and beta diversity measures were not affected by temperature or storage time (Fig. 3A–E). At the phylum level, the relative abundance of Firmicutes was greater (*p* = 0.026) in samples at baseline (24.80%) than in D1-37 (18.73%) and D1-50 (18.70%) samples. At the genus level, the relative abundance of *Ruminococcus* was greater (*p* = 0.004) in baseline samples (0.09%) than in D1-37 (0.04%), D3-37 (0.05%), D1-50 (0.05%), and D3-50 (0.05%) samples. No other differences to taxa were observed. Changes of relative abundance of genera were small as correlation coefficients between baseline and D1-37 (r = 0.972), D3-37 (r = 0.967), D1-50 (r = 0.981), and D3-50 (r = 0.976) were high (Fig. 3F–I).

### Microbiota profile of stabilized samples is largely unaffected by freeze–thaw cycles

**Figure 4:**
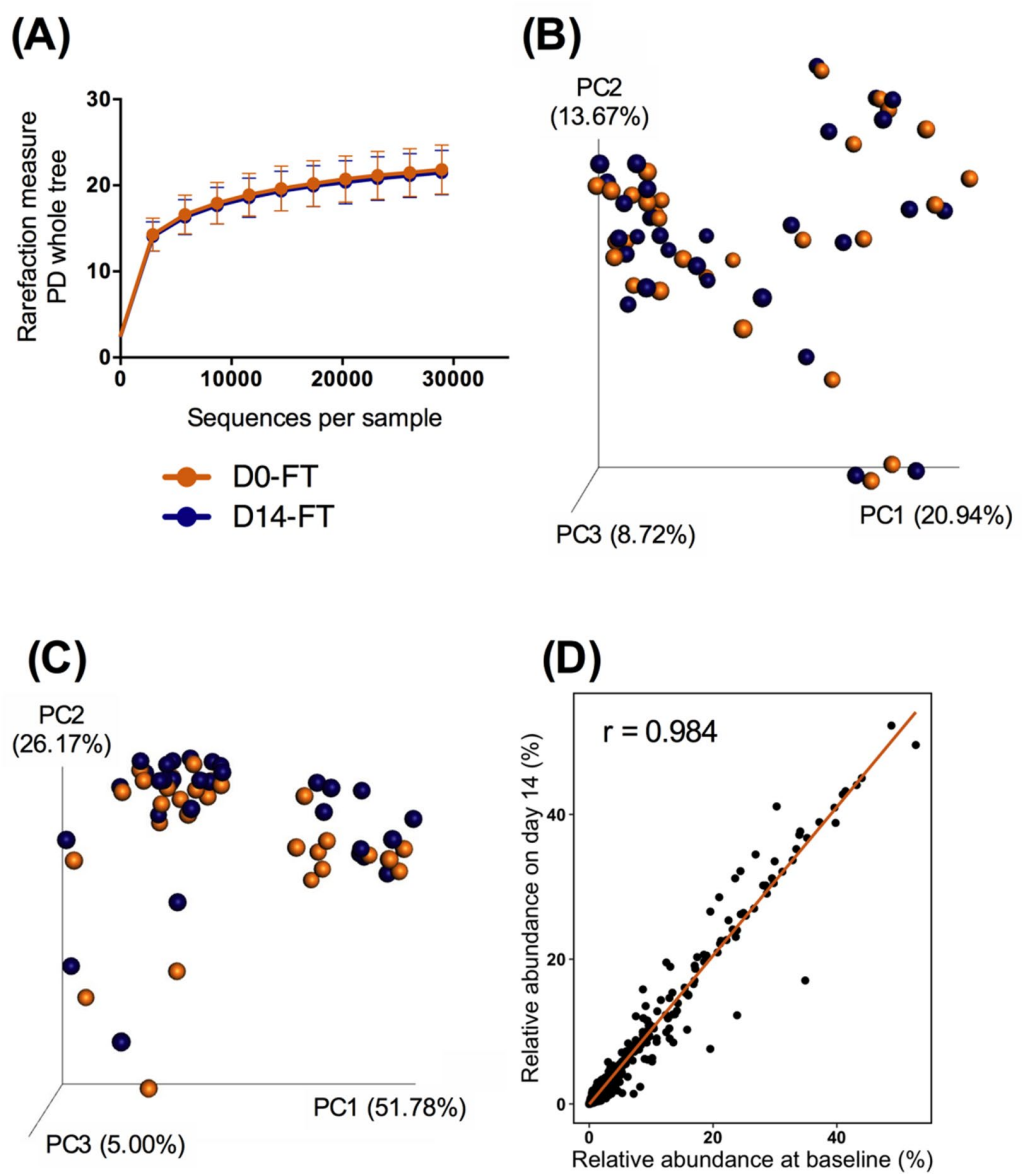
Fecal microbiota communities of stabilized samples at baseline (D0-FT) and stored at freeze–thaw cycles (D14-FT). (**A**) Alpha diversity measures, including phylogenetic diversity (PD) whole tree (shown), suggested that species richness and diversity were not affected by storage condition (ANOVA, *p* = 0.630). Principal coordinates analysis plots of unweighted (**B**) and weighted (**C**) UniFrac distances of fecal microbial communities revealed that beta diversity was not altered by storage condition. The scatterplot (**D**) shows the relative abundance of each genus in baseline samples against samples after freeze–thaw cycles.

Stabilized samples that underwent 6 freeze–thaw cycles (D14-FT) were compared with baseline samples (D0-RT) to evaluate the effectiveness of the device to stabilize samples undergoing temperature changes. An even sampling depth of 28,957 sequences per sample was used for assessing alpha- and beta diversity measures. Alpha- (*p* = 0.630) and beta diversity measures were not different between samples before and after the freeze–thaw cycles (Fig. 4A–C). Relative abundances of bacteria at the phylum level were not affected (*p* > 0.05) by freeze–thaw cycles. At the genus level, relative abundances of *Collinsella* were greater (*p* = 0.041; D0: 0.37%, D14: 0.68%), while relative abundances of *Faecalibacterium* (*p* = 0.010; D0: 1.13%; D14: 0.67%) and *Phascolarctobacterium* (*p* = 0.008; D0: 2.70%; D14: 1.83%) were lower in samples stored at freeze–thaw cycles for 14 days (D14-FT). No other differences to taxa were observed. Changes of relative abundance of genera were small as the correlation coefficient between baseline and D14-FT was high (r = 0.984; Fig. 4D).

### Stabilized samples yield consistent microbiota profiles, while unstabilized samples result in substantial changes in microbiota profiles when stored at room temperature

**Figure 5:**
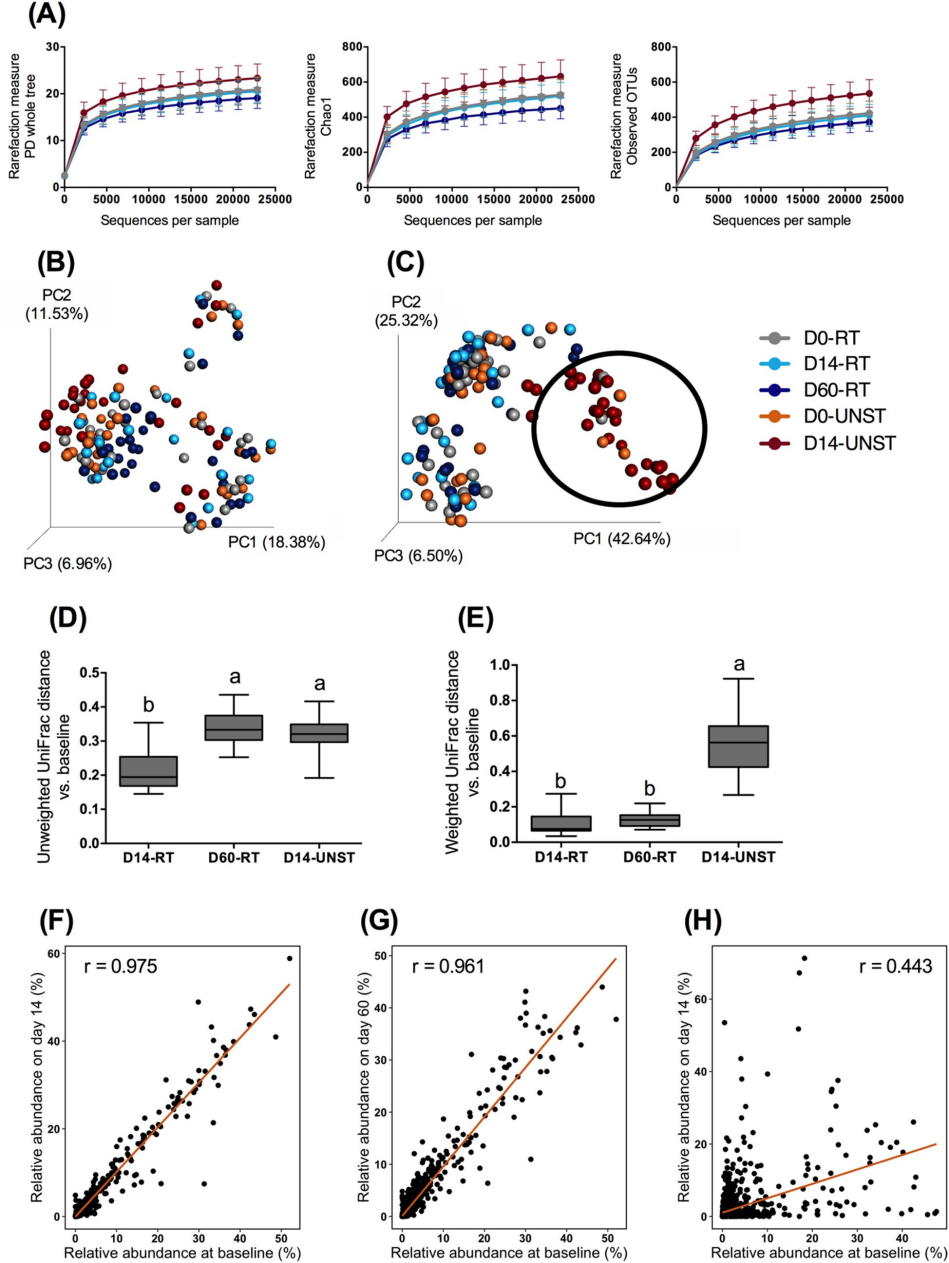
Fecal microbiota communities of stabilized and unstabilized samples at baseline (D0-RT, D0-UNST) and stored at room temperature (RT) for 14 (D14-RT, D14-UNST) and 60 days (D60-RT). (**A**) Alpha diversity measures, including phylogenetic diversity (PD) whole tree, Chao1, and observed operational taxonomic units (OTUs), suggested that species richness and diversity were greater in unstabilized samples stored at RT for 14 days (Tukey’s HSD, *p* < 0.001). The Chao1 metric showed a lower diversity in D60-RT than those of D0-RT (Tukey’s HSD, *p* = 0.0193), D14-RT (Tukey’s HSD, *p* = 0.0431), and D0-UNST (Tukey’s HSD, *p* = 0.013). Principal coordinates analysis (PCoA) plots of unweighted (**B**) UniFrac distances of fecal microbial communities revealed that beta diversity was not altered by storage condition. PCoA plots of weighted (**C**) UniFrac distance showed that D14-UNST samples clustered together (circled area) and away from other samples. Unweighted UniFrac distance from baseline (**D**) revealed that D14-UNST and D60-RT had greater distance than 14-RT. Weighted UniFrac distance (**E**) showed that D14-UNST had a greater distance than stabilized samples. ^a,b^ mean values with unlike letters were significantly different (Tukey’s HSD, *p* < 0.05). Scatterplots show the relative abundance of each genus in baseline samples against stabilized samples stored at RT for 14 (**F**) and 60 days (**G**), and unstabilized samples stored at RT for 14 days (**H**).

To investigate the ability of the device to stabilize fecal samples at ambient temperature, stabilized samples stored at RT for 14 (D14-RT) and 60 days (D60-RT) were compared with baseline samples (D0-RT) as well as unstabilized samples from baseline (D0-UNST) and those stored at RT for 14 days (D14-UNST). An even sampling depth of 22,875 sequences per sample was used for evaluating alpha- and beta diversity measures. Alpha-diversity measures (Fig. 5A), including phylogenic diversity whole tree (*p* < 0.001), Chao1 (*p* < 0.001) and observed OTU (operational taxonomic unit; *p* < 0.001) revealed higher species richness in unstabilized samples stored at room temperature for 14 days (D14-UNST) than in stabilized samples or baseline unstabilized samples. In addition, the Chao1 metric showed a lower diversity in D60-RT than those of D0-RT (*p* = 0.0193), D14-RT (*p* = 0.0431), and D0-UNST (*p* = 0.013; Fig. 5A). Beta diversity measures revealed a shift for unstabilized samples (Fig. 5B,C). Principal coordinates analysis (PCoA) plot of weighted UniFrac distance showed that D14-UNST samples clustered together and away from other samples (Fig. 5C). In addition, D14-UNST and D60-RT had a greater unweighted UniFrac distance from baseline than D14-RT (*p* < 0.001; Fig. 5D) and D14-UNST had a greater weighted UniFrac distance from baseline than stabilized samples (*p* < 0.001; Fig. 5E). Relative abundances of four dominant phyla were different between D14-UNST samples and stabilized samples: Actinobacteria (*p* < 0.001) and Firmicutes (*p* < 0.001) were greater, while Bacteroidetes (*p* < 0.001) and Fusobacteria were lower (*p* < 0.001) in D14-UNST samples than D0-UNST and stabilized samples (Table 1). Additionally, relative abundances of many dominant genera were different between D14-UNST samples and stabilized samples (Table 1). Changes of relative abundance of genera in stabilized samples after 14 days and 60 days at RT were small because the correlation coefficients between D0-RT and D14-RT (r = 0.975) and D60-RT (r = 0.961) were high (Fig. 5F,G). A great change was observed in unstabilized samples, where the correlation coefficient between D0-UNST and D14-UNST was r = 0.443 (Fig. 5H).

**Table 1:** Relative abundance of predominant bacterial phyla and genera in stabilized and unstabilized samples at baseline (D0) and stored at room temperature for 14 (D14) and 60 days (D60) (% of total sequences).

Phylum	Family	Genus*	Stabilized			Unstabilized		SEM	*P* value
			D0	D14	D60	D0	D14		
Actinobacteria			0.43^b^	0.49^b^	0.63^b^	0.42^b^	2.34^a^	0.44	< 0.001
	Bifidobacteriaceae	*Bifidobacterium*	0.08^b^	0.08^b^	0.03^b^	0.11^a,b^	0.63^a^	0.30	0.010
	Coriobacteriaceae	*Collinsella*	0.35^b^	0.41^b^	0.60^b^	0.30^b^	1.66^a^	0.32	< 0.001
		*Slackia*	0.01^b^	0.00^b^	0.00^b^	0.01^b^	0.03^a^	0.01	< 0.001
Bacteroidetes			38.79^a^	41.92^a^	41.34^a^	38.90^a^	8.34^b^	5.87	< 0.001
	Bacteroidaceae	*Bacteroides*	17.96^a^	18.71^a^	18.76^a^	17.07^a^	6.14^b^	3.62	< 0.001
	Prevotellaceae	*Prevotella*	11.86^a^	12.93^a^	12.13^a^	12.58^a^	0.38^b^	6.07	0.006
Firmicutes			24.63^b,c^	18.28^c^	20.16^b,c^	27.66^b^	72.55^a^	5.19	< 0.001
	Clostridiaceae	*Clostridium*	0.71^b^	0.60^b^	0.66^b^	1.09^b^	3.00^a^	0.46	< 0.001
		Unclassified	1.69	1.52	1.49	2.76	6.18^a^	0.86	< 0.001
	Enterococcaceae	*Enterococcus*	0.04^b^	0.03^b^	0.00^b^	0.07^a,b^	0.32^a^	0.15	0.010
	Erysipelotrichaceae	*Allobaculum*	0.28^b^	0.21^b^	0.28^b^	0.22^b^	1.22^a^	0.38	< 0.001
		*Catenibacterium*	1.04^b^	1.17^a,b^	0.96^b^	0.71^b^	3.96^a^	1.58	0.009
		*Coprobacillus*	0.06^b^	0.05^b^	0.03^b^	0.06^b^	0.33^a^	0.12	< 0.001
		Unclassified	0.19^b^	0.14^b^	0.11^b^	0.13^b^	0.78^a^	0.21	< 0.001
	Lachnospiraceae	*Blautia*	1.19^b^	1.11^b^	1.06^b^	1.03^b^	5.92^a^	0.74	< 0.001
		*Coprococcus*	0.03^b^	0.02^b^	0.02^b^	0.03^b^	0.15^a^	0.03	< 0.001
		*Dorea*	0.46^b^	0.32^b^	0.41^b^	0.43^b^	2.16^a^	0.39	< 0.001
		Unclassified	1.41^b^	1.16^b^	1.24^b^	1.22^b^	3.87^a^	0.43	< 0.001
	Lactobacillaceae	*Lactobacillus*	1.83^b^	1.49^b^	1.61^b^	3.35^b^	14.18^a^	3.95	< 0.001
	Peptococcaceae	*Peptococcus*	0.09^b^	0.07^b^	0.01^b^	0.12^b^	0.42^a^	0.10	< 0.001
	Peptostreptococcaceae	Unclassified	0.20^b^	0.20^b^	0.16^b^	0.23^b^	1.44^a^	0.30	< 0.001
	Ruminococcaceae	*Butyricicoccus*	0.01^b^	0.03^b^	0.02^b^	0.01^b^	0.09^a^	0.02	< 0.001
		*Faecalibacterium*	1.04^a,b^	0.68^b^	0.63^b^	0.56^b^	1.31^a^	0.30	< 0.001
		*Ruminococcus*	0.10^b^	0.09^b^	0.09^b^	0.06^b^	0.35^a^	0.09	< 0.001
		Unclassified	1.40^b,c^	1.04^b,c^	1.67^b^	0.88^c^	2.92^a^	0.39	< 0.001
	Streptococcaceae	*Streptococcus*	0.63^b^	0.39^b^	0.81^b^	0.97^a,b^	2.96^a^	1.18	0.007
	Turicibacteraceae	*Turicibacter*	1.07^b^	1.22^b^	1.06^b^	1.23^b^	4.15^a^	0.99	< 0.001
		*Phascolarctobacterium*	2.78^a^	1.90^a,b^	2.73^a^	1.14^b^	1.86^a,b^	0.52	< 0.001
Fusobacteria			27.58^a^	29.37^a^	27.72^a^	24.65^a^	10.14^b^	6.00	< 0.001
	Fusobacteriaceae	*Fusobacterium*	23.26^a^	24.63^a^	23.39^a^	20.58^a^	8.57^b^	5.06	< 0.001
		Unclassified	4.32^a^	4.74^a^	4.33^a^	4.07^a^	1.57^b^	1.22	< 0.001
Proteobacteria			8.39	9.74	9.97	8.23	6.22	2.90	0.277
	Alcaligenaceae	*Sutterella*	5.65^a^	6.29^a^	6.48^a^	6.14^a^	1.61^b^	0.99	< 0.001
	Succinivibrionaceae	*Anaerobiospirillum*	1.95^a,b^	2.54^a^	2.68^a^	1.61^a,b^	0.18^b^	1.25	0.018
Tenericutes			0.16^b^	0.18^a,b^	0.18^a,b^	0.12^b^	0.41^a^	0.13	< 0.001

^a,b,c^Mean values within a row with unlike superscript letters differ (Tukey’s HSD, *p* < 0.05).

*Genera with statistically different relative abundances among storage conditions are presented.

### Microbial composition varies among collection sites within a fecal sample

To evaluate the intra-sampling variation, we compared aliquots collected from both ends (Rep1, 3) and the middle (Rep2) of each fresh fecal sample. At the phylum level, relative abundance of Actinobacteria was different (*p* = 0.021) among collection sites. Relative abundances of several genera, including *Clostridium* (*p* = 0.016), *Collinsella* (*p* = 0.026), *Dialister* (*p* = 0.023), *Escherichia* (*p* = 0.045), *Faecalibacterium* (*p* = 0.011), *Phascolarctobacterium* (*p* = 0.001), *Roseburia* (*p* = 0.004), *Ruminococcus* (*p* < 0.001), *Sarcina* (*p* = 0.023), and *Shigella* (*p* = 0.019), were different among collection sites (Table 2).

**Table 2:** Relative abundance of predominant bacterial phyla and genera in samples collected from different sites (Rep1 and Rep1: two ends; Rep2: middle) of a single fecal sample (% of total sequences).

Phylum	Genus*	Rep 1	Rep 2	Rep 3	SEM	*P* value
Actinobacteria		0.59^a,b^	0.87^a^	0.46^b^	0.18	0.021
	*Collinsella*	0.368^a,b^	0.617^a^	0.340^b^	0.133	0.026
Bacteroidetes		29.34	25.28	28.42	3.31	0.308
Firmicutes		39.65	45.35	40.48	5.99	0.468
	*Clostridium*	1.074^b^	2.167^a^	1.622^a,b^	0.445	0.016
	*Dialister*	0.001^b^	0.004^a^	0.001^b^	0.002	0.023
	*Faecalibacterium*	0.900^a,b^	1.476^a^	0.764^b^	0.295	0.011
	*Phascolarctobacterium*	1.448^a,b^	2.514^a^	2.015^b^	0.334	0.001
	*Roseburia*	0.006^a^	0.003^a,b^	0.001^b^	0.002	0.004
	*Ruminococcus*	0.188^a^	0.062^b^	0.033^b^	0.048	< 0.001
	*Sarcina*	0.018^a^	0.000^b^	0.000^b^	0.009	0.023
Fusobacteira		23.99	20.67	24.85	4.67	0.526
Proteobacteria		6.34	7.71	5.61	1.10	0.071
	*Escherichia*	0.003^b^	0.003^b^	0.011^a^	0.005	0.045
	*Shigella*	0.010^a^	0.001^b^	0.000^b^	0.005	0.019

^a,b,c^Mean values within a row with unlike superscript letters differ (Tukey’s HSD, *p* < 0.05).

*Genera with statistically different relative abundances among collection sites are presented.

## Discussion

As the relationship between the gut microbiota and human health has become clearer, interest in the microbiome field has increased dramatically. A similar trend has been observed in companion animals, as pet owners are now considering their pets to be family members and prioritize health and longevity. One critical factor that contributes to microbiome data integrity and accuracy is sample collection and storage methods. Currently, rapid freezing and storage of fecal samples at − 80 °C is widely considered to be the gold standard. This method, however, is often challenging and costly, which limits the ability of collection at remote sites or collection of a large sample number. Therefore, an ambient collection and stabilization strategy would be of great value for such microbiome studies.

In this study, we collected and stored canine fecal samples using a commercially available device, PERFORMAbiome•GUT (DNA Genotek, Inc. Ottawa, Canada). Stabilized samples were incubated at different conditions to evaluate the ability of the stabilization device to preserve fecal samples for microbiota analysis. In accordance with previous studies using a similar device for humans^23,24,32^, our results revealed that alpha- (species richness and evenness within samples) and beta diversity (species richness among samples) were not altered in stabilized samples after storage at 37 °C and 50 °C for up to 3 days, at RT for up to 14 days, or undergoing 6 freeze–thaw cycles. In contrast, substantial changes were observed in the unstabilized samples, where alpha diversity increased, and the abundance of several taxa were altered after 14 days at RT. In support of our findings, shifted microbiota profiles were reported in unstabilized human samples after 48 or 72 hours at RT^16,23,33^.

For stabilized samples stored at RT for 60 days, the Chao1 index for alpha diversity measures was decreased compared with baseline samples and samples stored for 14 days. The Chao1 index is a metric highly favoring rare OTUs. The longer storage with the PERFORMAbiome•GUT kit may reduce the diversity by decreasing numbers of rare OTUs. However, the relative abundance of the main phyla and genera were not significantly different among stabilized samples stored at RT at any time points. Additionally, correlations of relative abundance of genera after 60 days against baseline were high (r = 0.961). This was also observed in a previous study where human and canine fecal samples were stored at RT for 56 days using the OMNIgene•GUT, and the correlations of OTU abundance against baseline samples were r = 0.96^32^.

Previous studies using OMNIgene•GUT kits for human fecal samples have reported changes in the relative abundance of a few genera. For example, a decreased abundance of *Sutterella* and *Faecalibacterium* were observed after 72 hours at RT^23^. Hill et al. noted the decreased abundance of *Clostridium* (XIVa and XVII) and *Sorobacter* as well as an increased abundance of *Faecalibacterium* after 1 week at RT^34^. Here, we found changes in *Faecalibacterium* and *Clostridium*, but not other genera reported in previous studies. After fecal samples were stabilized in the PERFORMAbiome•GUT device, an immediate increase in *Faecalibacterium* and decrease in *Clostridium* were noted when compared to the unstabilized samples. Additionally, the abundance of *Faecalibacterium* was decreased after six freeze–thaw cycles. This stabilization device and a similar product may not have the best ability to preserve *Faecalibacterium* as shown here and in two previous studies^23,34^. Therefore, this should be considered when testing samples of animals with gastrointestinal diseases, whereby *Faecalibacterium* abundance has been shown to be altered in both humans and dogs^13,35,36^.

Significant changes in the abundance of a few other taxa were observed in stabilized samples at high temperatures or freeze–thaw cycles. These changes, however, are considered relatively small when samples are stored at extreme conditions. In addition, correlations of genera abundance between baseline and after storage were high (r > 0.96). Similar to the results reported by Song et al. (2016) who used OMNIgene•GUT kits for human and dog fecal samples, small changes were noted in stabilized samples stored under two freeze–thaw (− 20 °C/RT) and two heat (4 °C/40 °C) cycles, as correlations between OTU abundance at baseline and after 8 weeks were high (r = 0.93 and 0.77, respectively). The unstabilized samples, on the other hand, showed large shifts after freeze–thaw or heat cycles (r = 0.15 and 0.41, respectively)^32^.

Finally, we showed the intra-sampling variation of the microbiota profile in this study. The relative abundance of nine genera were different among collection sites within a fecal sample. Gorzelak et al. also found high variation in microbial abundance when human fecal samples were not homogenized before DNA extraction^37^. Therefore, these data suggest that homogenization of fecal samples should be done so that a consistent microbial composition reflecting the entire fecal sample is reported.

Our findings demonstrate that the collection and storage strategy tested in this study minimized changes in microbiota profiles of canine fecal samples stored at ambient temperatures or undergoing significant temperature changes. This device should enable sample collection and storage when ultra-low temperature storage and transport methods are not feasible, allowing for collection at remote locations. This strategy also allows for shipment at low or high temperatures and long-term storage at ambient temperatures. Together, this device appears to be a robust approach for canine fecal sample collection and storage.

## Methods

### Fecal sample collection

All animal care and experimental procedures were approved by the University of Illinois Institutional Animal Care and Use Committee before experimentation (protocol #17008, #17135, and #17180). All methods were performed in accordance with the United States Public Health Service Policy on Humane Care and Use of Laboratory Animals. Fresh fecal samples were collected from 30 healthy female adult beagles (average age: 4.1 ± 1.3 years) within 15 minutes of defecation. All fecal samples were scored according to the following scale that has been used by our research group for decades^38,39^: 1 = hard, dry pellets, small hard mass; 2 = hard, well formed, dry stool; 3 = soft, formed, and moist stool, retains shape; 4 = soft, unformed stool, assumes shape of container; and 5 = watery, liquid that can be poured. All fecal samples had scores ranged from 2.5–3, which are considered to be normal, healthy stools. Three subsamples (2 ml; from each end and middle of a fecal sample) were collected and placed into cryovials and immediately frozen and stored at − 80 °C. The remaining fecal samples were aliquoted either into a conical tube stored at room temperature or into three PERFORMAbiome•GUT collection devices (PB-200) (DNA Genotek, Inc. Ottawa, Canada). After baseline microbiota data were obtained from each collection device, one stabilized sample was aliquoted and incubated at 37 °C and 50 °C. The other two stabilized samples were stored at RT (~ 23 °C) or undergoing six freeze–thaw (− 20 °C/30 °C) cycles (Fig. 1).

### Fecal DNA extraction, amplification, and sequencing

Total bacterial DNA of all fecal samples was extracted according to the timeline shown in Fig. 1. Baseline samples were extracted on the collection day within 2 hours of collection. Total DNA was extracted according to manufacturer’s instructions using the MO BIO PowerFecal Kit (MO BIO Laboratories, Carlsbad, CA) with bead beating using a vortex adaptor. Concentration of extracted DNA was quantified using a Qubit® 3.0 Fluorometer (Life Technologies, Grand Island, NY). Following extraction, the V3-V4 region of the bacterial 16S rRNA gene was amplified by polymerase chain reaction using the following forward and reverse primers: Bakt_341F (CCTACGGGNGGCWGCAG) and Bakt_805R (GACTACHVGGGTATCTAATCC), which include an additional 0–7 bases between the transposase and primer sequences, and paired such that the final combination of primer pairs sum to the same length. 16S rRNA template amplification and index addition was performed using Illumina’s recommended 16S library preparation protocol with a customized dual index version of Illumina’s Nextera XT indexing protocol. Amplicons were sequenced on an Illumina MiSeq using 2 × 300 bp PE MiSeq reagent kit V3. Base calling diversity for amplicon sequencing was increased through use of 10% PhiX spike into each sequencing pool and through variable length spacers in primer sequences.

### Bioinformatics and statistical analysis

Trimmomatic was used to remove sequencing adaptors and low-quality reads^40^. The FLASH algorithm was used for read merging and automated rejection of low quality sequences^41^. Quality screening for length and ambiguous bases was performed with proprietary scripts. Quantitative Insights Into Microbial Ecology (QIIME 1.9.1)^42^ was used to process the sequence data. A closed-reference taxonomic classification was performed, where each sequence was aligned to the curated SILVA version 123 reference database^43^. Sequences were aligned at 97% sequence identity using the NINJA-OPS algorithm, version 1.5.1^44^. At 97% sequence identity, each OTU represents a genetically unique group of biological organisms. These OTUs were then assigned a curated taxonomic label based on the seven-level SILVA taxonomy. Alpha- and beta diversity measures were assessed at an even sampling depth sequences per sample. Data for each specific comparison (i.e., research aim) were analyzed separately. Alpha diversity was estimated using phylogenetic diversity whole tree, Chao1, and observed OTU matrices. Beta diversity was calculated using weighted and unweighted UniFrac^45^ distance measures, and presented as PCoA plots. UniFrac distances between baseline and samples stored at 37 °C and 50 °C as well as baseline and samples stored at RT were calculated and compared. Correlation of relative abundance of genera between baseline and samples after incubations were analyzed using Pearson’s correlation. Statistical analysis was conducted via Statistical Analyses of Metagenomic Profiles (STAMP) software 2.1.3^46^ and SAS 9.4 using ANOVA and Tukey–Kramer multiple comparison tests. All tests were corrected for multiple inferences using the Benjamini–Hochberg method to control for false discovery rate^47^. Statistical significance was set at *p* < 0.05.
